# Controllable one-pot synthesis of uniform colloidal TiO_2_ particles in a mixed solvent solution for photocatalysis

**DOI:** 10.3762/bjnano.9.163

**Published:** 2018-06-08

**Authors:** Jong Tae Moon, Seung Ki Lee, Ji Bong Joo

**Affiliations:** 1 Department of Chemical Engineering, Konkuk University, 120 Neungdong-ro, Gwangjin-gu, Seoul 05029, Republic of Korea; 2 Department of Life Science, University of Seoul, 163 Seoul Siripdae-ro, Dondaemun-gu, Seoul 02504, Republic of Korea

**Keywords:** mixed solvent, one-pot synthesis, photocatalysts, rhodamine B degradation, sol–gel synthesis, spherical TiO_2_ particles

## Abstract

This study reports on the controllable synthesis of uniform colloidal titanium dioxide (TiO_2_) particles and their photocatalytic applications toward rhodamine B (RhB) degradation. The monodispersed TiO_2_ particles were synthesized under mixed solvent conditions by sol–gel chemistry in a one-pot process. Varying the ratio of solvent composition, the concentration of surfactant and TiO_2_ precursor was used to control the particle diameter, degree of monodispersity and morphology. The modification of the calcination temperature affected the crystallinity and crystalline phase of the colloidal TiO_2_ particles. When uniform, amorphous TiO_2_ particles were calcined at an optimal temperature (500 °C), the final sample exhibited beneficial characteristics such as high anatase crystallinity with a mixed phase of anatase and rutile and relatively high surface area. The photocatalytic efficiency of the uniform TiO_2_ sample with high anatase crystallinity with mixed phase and high surface area was dramatically enhanced towards RhB degradation under UV–vis irradiation. We systemically discuss the relationship between the synthetic parameters in our synthesis and the properties of the final TiO_2_ products, as well as the crystalline properties and performance enhancement of TiO_2_ photocatalysts calcined at different temperatures.

## Introduction

Titanium dioxide (TiO_2_) is a widely explored semiconducting material that exists in abundance from natural resources. It has a wide range of applications including bio-separation, sensors, energy storage, catalysis and photocatalysis [1–11]. Due to its wide band gap energy (3.0–3.2 eV) between the conduction and valance bands, TiO_2_ can absorb photons in the ultraviolet (UV) portion of the light spectrum. This leads to the sequential generation of electron–hole pairs that can induce a variety of surface redox reactions. Photocatalytic water splitting via a TiO_2_ electrode under UV irradiation was first reported by Honda and Fujishima [12]. Over the past decade, there have been many attempts to fabricate highly active TiO_2_-based photocatalysts for enhanced photocatalytic efficiency [13–17].

It is well-known that photocatalysis over a semiconductor photocatalyst occurs in the following sequential steps. First, the semiconductor photocatalyst can absorb photon energy that is greater than its band gap and electrons in the valance band can be exited to the conduction band, resulting in photoexcited electron–hole pairs. Then, the photo-exited electron–hole can move to the surface of the photocatalyst without charge recombination. Finally, each charge carrier can be consumed for surface redox reactions [9,15]. To achieve high performance in the overall photocatalysis system, the efficiency of each step should be improved [13]. Regarding the point of light absorption, the band gap energy range of the photocatalyst must be narrow in order to facilitate the facile adsorption of the low energy photon and high harvesting efficiency under visible-light irradiation conditions [11]. To enhance charge separation efficiency and extend the lifetime of photoexcited electron–hole pairs, properties such as a crystalline phase or high crystallinity are essential. Highly crystallized TiO_2_ is generally considered to not only reduce the recombination of electron–hole pairs, but to also extend the lifetime of photogenerated charges that result in the enhancement of photocatalytic activity [7]. During photocatalysis, it is well-known that photo-generated electrons can transfer to the bulk in a TiO_2_ crystallite, while the hole can move towards the interface between the TiO_2_ and solution [18–19]. Once the TiO_2_ has a well-crystallized structure, electrons can easily move toward the bulk and freely exist in the bulk of the crystallite, which results in a charge delocalization that leads to a decreasing chance of electron–hole recombination [19]. In addition, even though the well-crystallized anatase phase is superior for photocatalysis under UV conditions due to its intrinsic properties (e.g., low recombination rate of photoexcited electron–hole pairs), it is also well-known that a mixed anatase and rutile crystalline phase can lead to enhanced photocatalysis performance. In particular, the exceptional activity of commercial P25 TiO_2_ is often explained to originate from its unique crystalline properties, i.e., its mixed anatase/rutile phase with high anatase crystallinity. As shown in both a previous study and our later discussion, a mixed crystalline phase of anatase and rutile in P25 TiO_2_ can have several beneficial effects such as improved light adsorption in the low energy UV range and separation of photoexcited charge carriers which can result in significantly enhanced photocatalysis performance [20–21]. From the surface reaction point of view, a large surface area of the TiO_2_ photocatalyst is important for increasing the number density of redox reaction sites [22]. Thus, it is critically essential to synthesize TiO_2_ photocatalysts with controlled crystallinity while maintaining a high surface area.

Since many photocatalysis reactions are a liquid phase reaction, a well-dispersible colloidal TiO_2_ nanostructure should be one of the most ideal forms. In order to maximize the active surface area as well as improve dispersion in a reactant solution, one can consider the direct use of TiO_2_ nanocrystals [23–24]. However, nanocrystals synthesized by a conventional solution-based method are strongly stabilized with organic capping agents, which limit the accessibility of the reactant molecule to the nanocrystal surface and prevent surface reactions, and in turn, hamper the photocatalytic activity [25]. In order to address the above issues, TiO_2_ photocatalysts are often synthesized in the form of a porous colloidal particle in sub-micrometer dimensions [8,26–27]. They can be calcined at high temperature, which results in well-developed crystalline properties and clean active sites with a suitable high surface area. Sub-micrometer photocatalysts are usually stabilized by electrostatic charges from their surface so that no additional stabilizers are necessary for maintaining dispersion stability in a reaction solution [8]. In addition, the relatively larger size of the sub-micrometer photocatalysts compared to typical nanocrystal forms makes them easier to recycle from the reaction solution through simple centrifugation.

With continued development of synthetic chemistry, a variety of methods was suggested for preparing colloidal nanostructures and many colloidal TiO_2_ porous particles have been demonstrated [13,15,26]. Among the various colloidal TiO_2_ nanostructures, there has been increasing interest in uniform, colloidal TiO_2_ spheres due to their advantageous characteristics including facile functionalization, large surface area, uniform photocatalytic activity for each particle, and improved accessibility for the reactant molecules. To fabricate the uniform, colloidal TiO_2_ spheres, several synthetic methods have been reported including hydrothermal, solvothermal, and sol–gel processes [10,26,28–29]. Although hydrothermal or solvothermal process are simple and convenient, they are typically conducted under high pressure and high temperature conditions for an elongated reaction time in a sealed reactor which requires high energy and large expense. In addition, it is difficult to monitor the overall synthetic progress of the formation and growth of the particle and to control the properties of the final products. On the other hand, the sol–gel process, which is carried out under atmospheric pressure conditions at near room temperature, is well known as a suitable method for producing colloidal metal oxide particles, such as SiO_2_, ZrO_2_ and TiO_2_ that have well-controlled characteristics [15,30–31].

We have previously reported a reproducible sol–gel coating method for producing SiO_2_@TiO_2_ core–shell nanostructures in the presence of a surfactant in pure ethanol solution [7–8]. In addition, another robust sol–gel coating process in a mixed solvent of ethanol–acetonitrile (EtOH/ACN) for producing polymer@TiO_2_ core–shell nanostructures was reported [14]. Since the solubility of the TiO_2_ precursor, titanium butoxide (TBOT), is different in the two different solvents (ethanol and acetonitrile), the nucleation/growth rate of TBOT and its hydrolysate is finely controlled by regulating the ethanol–acetonitrile ratio. The spherical curvature and smoothness of the TiO_2_ layers was also tuned using the ethanol–acetonitrile solvent conditions that resulted from the controlled diffusion rate of TBOT and its hydrolysate in a mixed solvent [14,32–33]. Without a sacrificial core material, it should be noted that this sol–gel strategy allows the colloidal TiO_2_ particles to have finely tuned properties due to the well-controlled nucleation/growth rate of TBOT in mixed solvent conditions. Although this sol–gel coating strategy using a mixed solvent was successfully applied for synthesizing core–shell nanostructures, to the best of our knowledge, there is no obvious report on sol–gel synthesis of uniform colloidal metal oxide particles such as TiO_2_ in a mixed solvent solution of ethanol–acetonitrile.

In this study, the synthesis of uniform TiO_2_ colloidal particles with controllable properties and their photocatalytic application towards the degradation of rhodamine B is discussed. More specifically, uniform TiO_2_ colloidal particles were synthesized by a sol–gel process in a well-controlled mixed solvent of ethanol and acetonitrile, and the relationship between the synthetic parameters and the properties of the final TiO_2_ products were systemically studied. The resulting TiO_2_ particles exhibited beneficial properties towards the liquid phase photocatalysis, including uniform particle dimensions, well-developed mesoporous structure, and a mixed crystalline phase of anatase and rutile. The final uniform TiO_2_ particles displayed significantly improved photocatalytic activity. By controlling the calcination conditions, the crystalline phase and crystallinity of the uniform TiO_2_ particles can be fine-tuned and the catalyst activity can be optimized for further enhanced activity. To the best of our knowledge, this is first report on the synthesis and systemic study of uniform colloidal TiO_2_ particles using sol–gel synthesis in mixed solvent conditions using ethanol–acetonitrile.

## Results and Discussion

**Scheme 1:**
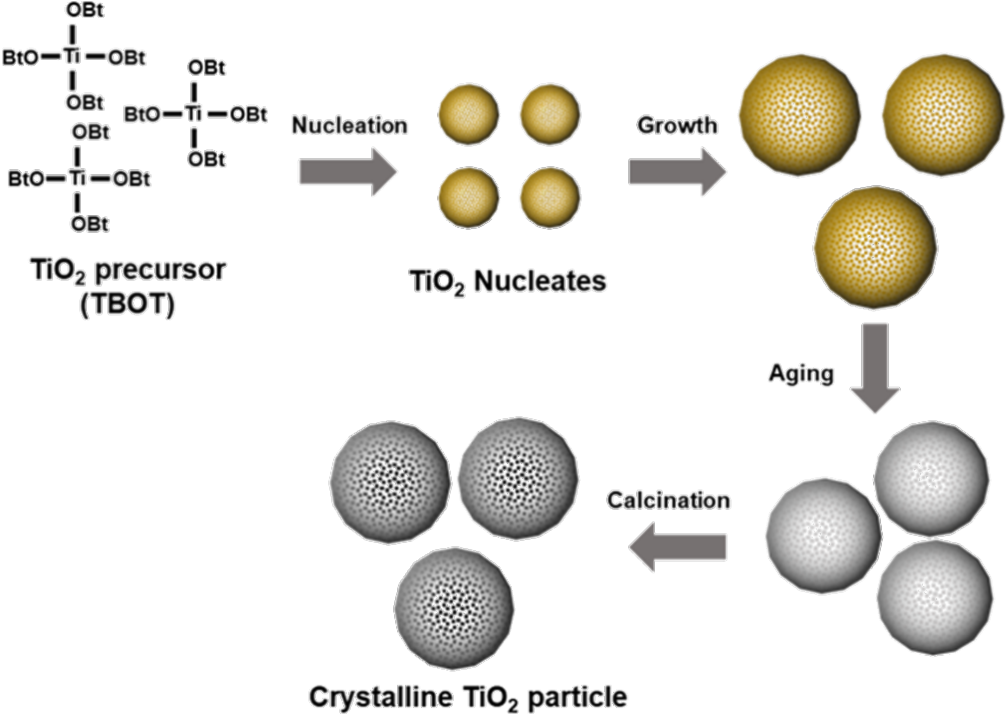
Schematic illustration of the synthesis of uniform TiO_2_ particles using a sol–gel reaction in mixed solvent conditions.

Uniform colloidal TiO_2_ particles can be synthesized by a modified sol–gel synthesis followed by calcination under atmospheric conditions. The synthesis is conducted in the ethanol–acetonitrile mixed solvent phase in the presence of a surfactant and base catalysts for the hydrolysis and condensation of the TiO_2_ precursor. Specifically, a typical synthesis involves three steps, as illustrated in Scheme 1: 1) the formation of TiO_2_ nucleates and subsequent growth of amorphous TiO_2_ spheres by a sol–gel reaction of titanium butoxide (TBOT) in the mixed solvent with hydroxypropylcellulose (HPC) as a surfactant; 2) aging step of the spherical TiO_2_ particles in the water-containing solvent to make the surface of TiO_2_ particles condensed; and 3) calcination to crystallize amorphous TiO_2_ particles into their crystallized counterparts.

The hydrolysis and condensation of the TBOT precursor are highly influenced by several factors such as the solvent environment, concentration of surfactant, and the amount of precursor used [13–15,31]. The precise tuning of these factors allows the convenient control of the physical–chemical properties of the final colloidal TiO_2_ particles such as particle size and macroscopic morphology. In this study, we optimized these factors to control the size of the TiO_2_ particles and prevent particle agglomeration.

**Figure 1:**
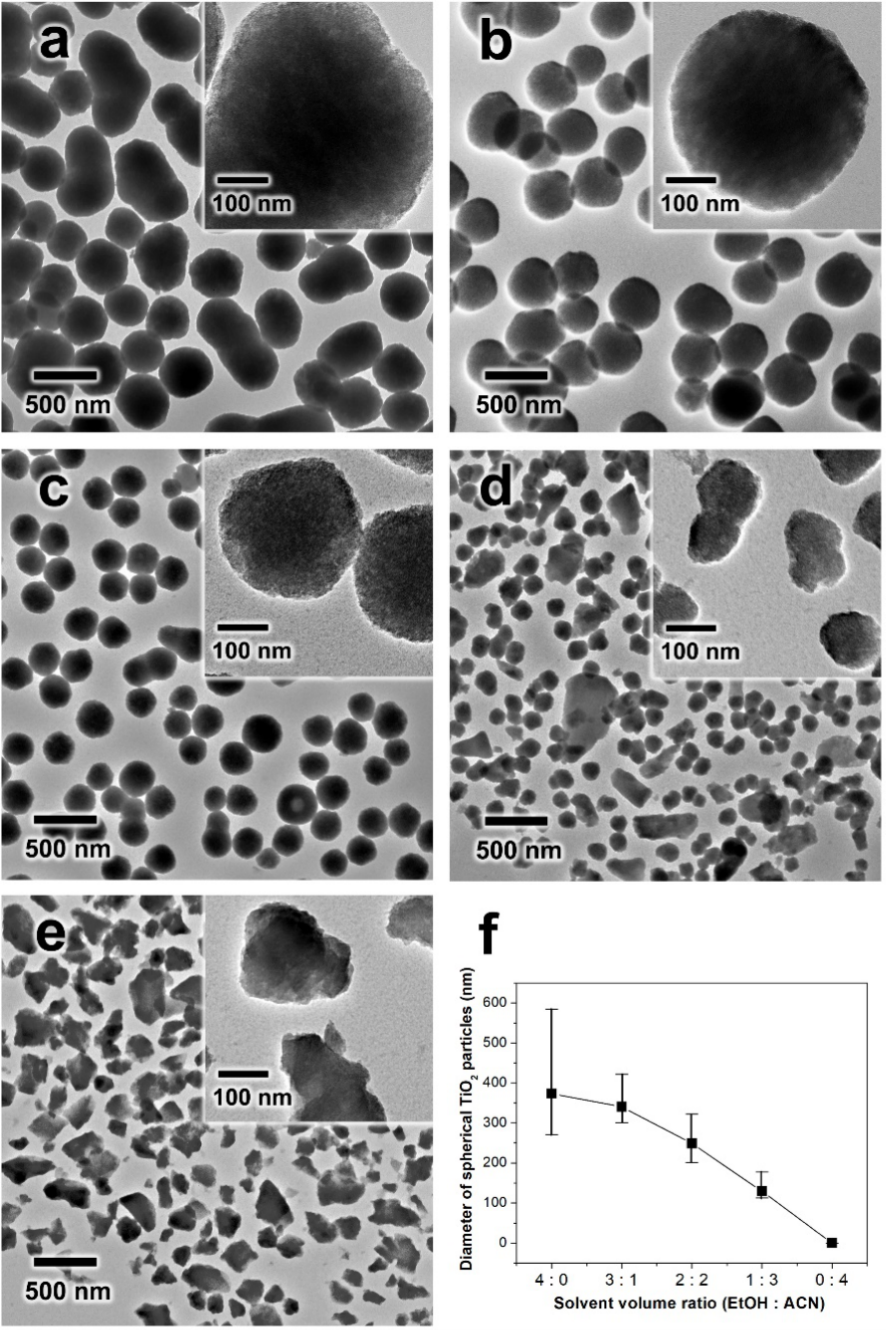
(a–e) TEM images of as-synthesized TiO_2_ with different volume ratios of ethanol to acetonitrile used as the solvent: (a) ethanol/ACN 4:0 (b) ethanol/ACN 3:1 (c) ethanol/ACN 2:2 (d) ethanol/ACN 1:3 and (e) ethanol/ACN 0:4. (f) The relationship between the solvent volume ratio and the average diameter of the spherical TiO_2_ particles.

Figure 1 compares the as-synthesized amorphous TiO_2_ particles prepared by varying the solvent composition. As shown in Figure 1a, when only pure ethanol was used as the solvent, the resulting TiO_2_ sample exhibited large TiO_2_ particles (≈400–500 nm) and the particles were quite aggregated. With the addition of acetonitrile into the synthesis, the particle size and tendency for particle aggregation dramatically changed. When the volume ratio of EtOH/ACN was 3:1, the particle diameter decreased to ≈340 nm and most of the particles were individually isolated without particle agglomeration (Figure 1b). When the volume ratio of EtOH/ACN was 2:2, the particle size of the spherical TiO_2_ particles became even smaller, reducing to ≈250 nm without apparent particle aggregation (Figure 1c). As the volume ratio of EtOH/ACN was decreased to 1:3, the particle diameter dramatically decreased and the shape of the TiO_2_ particles became irregular (Figure 1d). When pure ACN was used as a solvent, the resulting TiO_2_ sample displayed irregular particles without spherical shape. In addition, the particle size of the sample was not uniform and the surface of the particle was not smooth (Figure 1e). As shown in Figure 1f, the particle diameter of the spherical TiO_2_ sample continuously decreased from 380 to 340, 250, 130 and 0 nm with the change in the solvent volume ratio of EtOH/ACN from 4:0 to 3:1, 2:2, 1:3 and 0:4, respectively. A reported in other works, the solvent environment can significantly affect the particle size and morphology of the TiO_2_ sample [14,32]. As the ACN ratio increased, it was noted that the particle diameter of the spherical TiO_2_ continuously decreased and the particle shape became irregular.

These phenomena can be explained by not only the solubility of TBOT and the surfactant, but also by the nucleation-growth kinetics of TBOT in different solvent conditions. It is well-known that acetonitrile is more hydrophobic than ethanol [14]. As the relative ACN ratio increased in the mixed solvent, the precursor (TBOT) and surfactant (HPC), which have many hydrophilic hydroxy groups, were not stable in the mixed solvent. TBOT is not miscible and results in a separated phase, and HPC does not dissolve in the ACN solution. HPC (10 mg) could not be dissolved in ACN solution (10 mL) in the range of solvent conditions used in our works (Supporting Information File 1, Figure S1a). However, HPC (10 mg) can completely be dissolved in 10 mL of ethanol (Supporting Information File 1, Figure S1b). In addition, when we tried to dissolve TBOT in pure acetonitrile solution, TBOT (5 mL) and ACN (5 mL) cannot be mixed, resulting in a separated phase, as shown in Supporting Information File 1, Figure S1c. When TBOT (5 mL) is mixed with ethanol (5 mL), it shows the homogeneously mixed solution (Supporting Information File 1, Figure S1d).

According to a previous study, TBOT molecules have a high solubility in ethanol but not in acetonitrile [14]. As a result, when the concentration of ACN increases, the TBOT and its hydrolysate molecules are forced to form nucleates rapidly and to precipitate out of the solution [32–33]. This rapid nucleation and precipitation induces the formation of relatively small particles in the ACN-rich solvent. In addition, since the HPC was not dissolved and existed in the form of an undissolved solid in the ACN-rich solvent, the HPC molecules could not be positioned on the interface between the surface of the particle and the solvent. As a result, the HPC molecules could not interact with either the surface of the TiO_2_ particles or hydrolyzed TiO_2_ species (e.g., TBOT hydrolysate) in the ACN-rich solvent. Thus, it neither exhibited a stabilization effect on the TBOT hydrolysate nor any steric hindrance on the surface of the TiO_2_ particles, which resulted in the rapid formation of irregular, small TiO_2_ particles.

In contrast, when ethanol was used as a solvent, both TBOT and HPC were easily dissolved. Since the HPC surfactant contains many hydroxy and ether groups which can form hydrogen bonds with not only the hydroxy group of TBOT hydrolysate but also those of the TiO_2_ surface, the hydrolyzed TiO_2_ species should have been relatively stable in the ethanol solvent. In addition, the HPC molecules could also be positioned on the surface of the TiO_2_ particle, which displayed enough steric hindrance effects on the deposition of the hydrolyzed TiO_2_ species. Thus, the deposition kinetics of the hydrolyzed TiO_2_ species was controlled and the growth of the TiO_2_ particles preferentially occurred by the deposition of the hydrolyzed TiO_2_ species once the TiO_2_ nucleates were formed, which resulted in the formation of large TiO_2_ particles with a spherical curvature.

**Figure 2:**
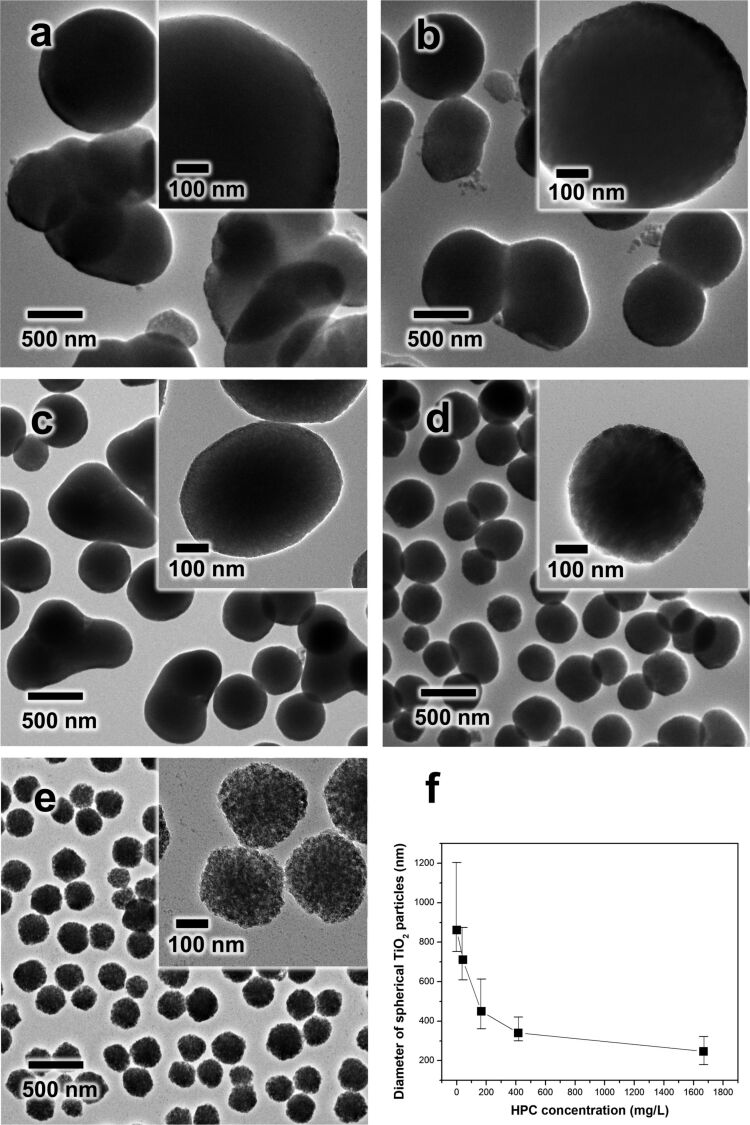
(a–e) TEM images of as-synthesized TiO_2_ with different concentrations of HPC: (a) 0 (b) 41.67 (c) 166.67 (d) 416.67 and (e) 1666.67 mg/L. (f) The relationship between HPC concetnration and average diameter of spherical TiO_2_ particles.

When a certain amount of ACN was added to the ethanol to form EtOH/ACN mixed solvent conditions, the TBOT molecules and their hydrolysates were less stable compared to the case of the pure ethanol solvent, and they were likely to precipitate [14,33]. The TBOT molecules and their hydrolysates formed the self-nucleated TiO_2_ particles, which could be grown by the controlled diffusion and deposition of TBOT and their hydrolysates on the surface of the TiO_2_ particles in the mixed solvent conditions. In addition, since HPC could also be dissolved in the mixed solvent, the dissolved HPC molecules not only tuned the stability of the TBOT molecules and their hydrolysates but also controlled deposition kinetics. The HPC molecules should also be positioned on the surface of the nucleated TiO_2_ particles, which resulted in the controlled deposition rate of the TBOT molecules and their hydrolysates, adjusted growth kinetics of the TiO_2_ particles, and prevention of particle aggregation. Thus, as-synthesized TiO_2_ particles, which were synthesized in the EtOH/ACN mixed solvent, displayed uniform spherical morphology with isolated particles. Since the TiO_2_ particles prepared under mixed solvent conditions (EtOH/ACN 3:1) showed great uniformity in size and morphology, the sample in Figure 2b was used for further control. The subsequent discussion will focus on the sample prepared in mixed solvent conditions with a ratio of EtOH/ACN of 3:1.

As mentioned in the previous section, the hydrolysis and condensation of the TBOT precursor were highly influenced by the concentration of HPC as a surfactant. Figure 2 compares the morphology of the TiO_2_ particles synthesized by using different concentrations of HPC in the mixed solvent conditions (EtOH/ACN 3:1). As shown in Figure 2a, without HPC, the as-synthesized TiO_2_ particles were severely aggregated with large particles. With an increased concentration of HPC, the tendency for particle aggregation dramatically decreased and the size of the primary TiO_2_ particle was reduced (Figure 2b,c). When the concentration of HPC was increased to 416.67 mg/L, isolated TiO_2_ particles were eventually produced and the particle size decreased to 340 nm (Figure 2d). As the concentration of HPC was increased further (1666.67 mg/L), the particle size became even smaller (250 nm), as shown in Figure 2e. The average diameter of the TiO_2_ particles was estimated to be approximately 860, 710, 450, 340 and 250 nm for the samples prepared with 0, 41.67, 166.67, 416.67, and 1666.67 mg/L of HPC, respectively (Figure 2f). As shown in both the previous literature and this study, the surfactant molecules (e.g., HPC) which contain many hydroxyl and ether groups create a strong interaction with the TiO_2_ surface via hydrogen bonding [9,31]. In addition, the TBOT hydrolysates were stabilized by the surfactant, which resulted in the controlled deposition on the growing TiO_2_ surface and produced small particles. Moreover, the binding of HPC prevented close contact the TiO_2_ particles, and therefore, suppressed interparticle agglomeration.

**Figure 3:**
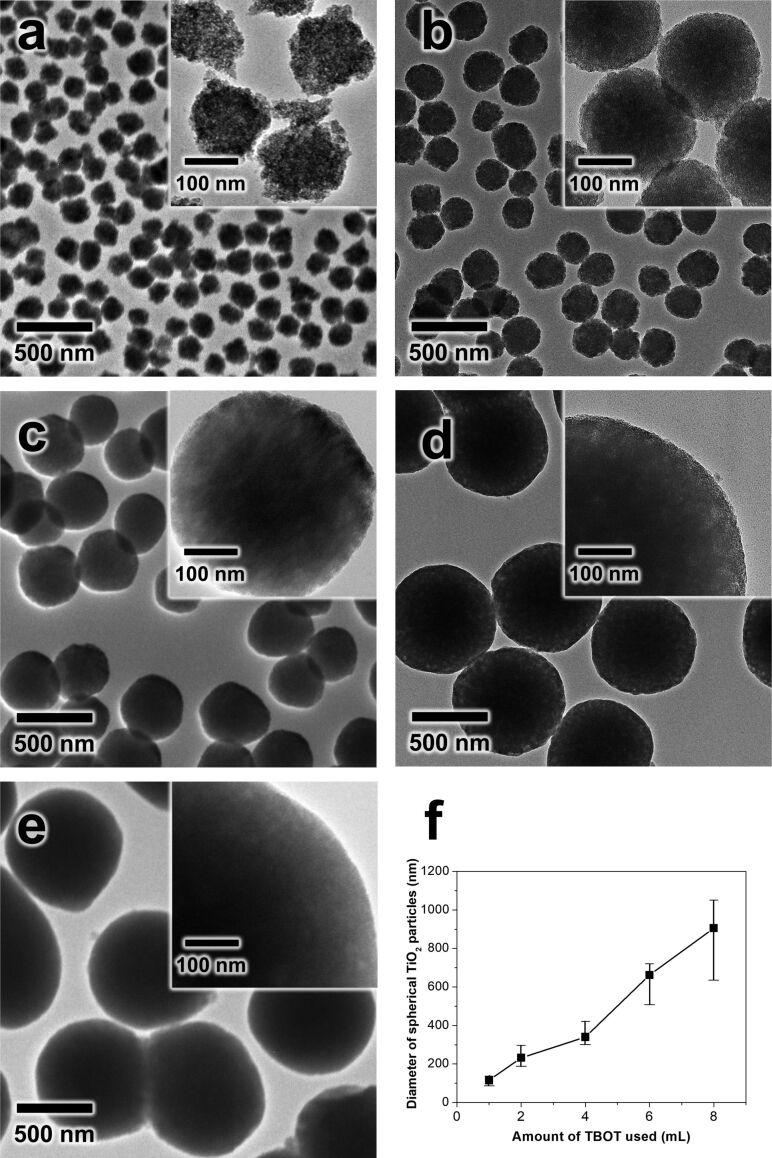
(a–e) TEM images of as-synthesized TiO_2_ with different amounts of TBOT: (a) 1 (b) 2 (c) 4 (d) 6 and (e) 8 mL. (f) The relationship between the amount of TBOT used and the average diameter of the spherical TiO_2_ particles.

Consistent with other syntheses, synthesizing uniform TiO_2_ particles was also controlled by changing the concentration of the precursor [14,31]. When 1 mL of TBOT was used, the as-synthesized TiO_2_ particles formed as small particles of about 110–120 nm diameter with a rough surface. As the concentration of the TBOT was increased, the particle size continuously increased (Figure 3b–e). The average diameter of the TiO_2_ particle was estimated to be approximately 120, 230, 340, 660, and 900 nm for the samples prepared using 1, 2, 4, 6, and 8 mL of TBOT, respectively (Figure 3f). Based on these results, we can control the monodispersity and uniformity of TiO_2_ particles by tuning either the solvent composition, concentration of surfactant or concentration of TBOT. To confirm the particle size and uniformity of as-synthesized TiO_2_, we conducted SEM under low magnification and dynamic light scattering (DLS) analysis. Supporting Information File 1, Figure S2 shows low magnification SEM images and the DLS results of as-synthesized amorphous TiO_2_ particles which were prepared by using a volume ratio of EtOH/ACN of 3:1, and calcination at 500 °C (labeled as TiO_2_-500). Even though the particle diameter is reduced due to the calcination at 500 °C, the spherical morphology of the particle is well maintained with uniform size (Supporting Information File 1, Figure S2a,b). In addition, DLS data showed that the amorphous TiO_2_ sample presented in Figure 1b displayed a sharp particle distribution peak centered at ≈350 nm. The calculated particle diameter of the TiO_2_ sample is ≈347 ± 75 nm, which is similar to the SEM results. TiO_2_-500 samples calcined at 500 °C also showed a sharp particle distribution peak at ≈262 ± 7 nm. Based on the SEM and DLS results, it can be concluded that our TiO_2_ particles are uniform (Supporting Information File 1, Figure S2c and inset table). To crystallize the TiO_2_ particles and improve the photocatalysis activity, the TiO_2_ particles were subjected to calcination at different temperatures. Because the sample shown in Figure 3c displayed a suitable particle size, morphological uniformity and no aggregation, we focus the subsequent discussion by using the TiO_2_ sample and its calcined derivatives.

**Figure 4:**
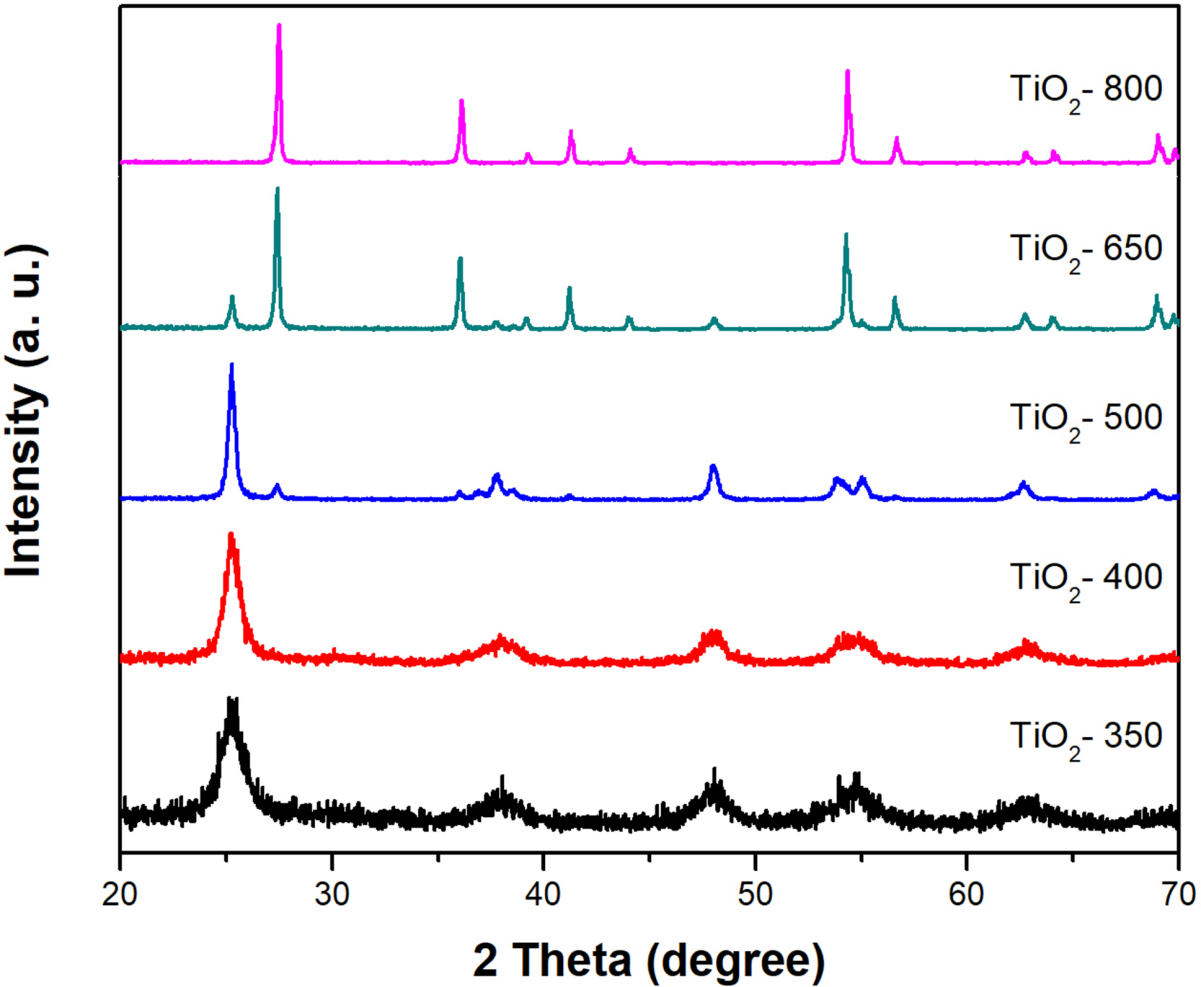
X-ray diffraction patterns of TiO_2_ particles calcined at different temperatures (≈350–800 °C).

The crystalline characteristics of the TiO_2_ particles calcined at different temperatures were investigated by X-ray diffraction (XRD). As shown in Figure 4, the TiO_2_ sample calcined at 350 °C (TiO_2_-350) showed relatively broad diffraction peaks at 2θ = 25.4, 37.9, 48.1, 54 and 55° which were attributed to the (101), (004), (200), (105) and (211) planes, respectively, indicating an anatase phase. As the calcination temperature increased, the peak sharpness increased, indicating enhanced crystallinity. The TiO_2_ sample calcined at 500 °C (TiO_2_-500) exhibited not only the obvious anatase peaks but also new peaks at 2θ = 27.6, 36.6, 38.0, 41.4 and 54.5° corresponding to the (110), (101), (111), (210) and (211) planes of the rutile TiO_2_ phase, respectively. When the sample was calcined at an even higher temperature of 650 °C (TiO_2_-650), the peaks related to the rutile phase were more obvious. In addition, the relative intensity of the rutile phase was much stronger than that of the anatase phase. Upon further increases of the calcination temperature to over 800°C, all of anatase peaks disappeared and only the strong peaks related to the rutile phase were observed, which indicated that the meta-stable anatase phase was completely converted to rutile by the thermal phase transformation. The average anatase crystallite sizes of the TiO_2_ samples, which were determined from the widths of the anatase (101) peaks in the XRD results by using the Scherrer equation, were estimated to be approximately 7, 10, 20, 29.7 and 0 nm for samples calcined at 350, 400, 500, 650, and 800 °C, respectively. In addition, the rutile grain sizes of the TiO_2_ samples calcined at 500, 650, and 800 °C were calculated as approximately 26, 45, and 48 nm, respectively, by using the rutile (110) peaks.

**Figure 5:**
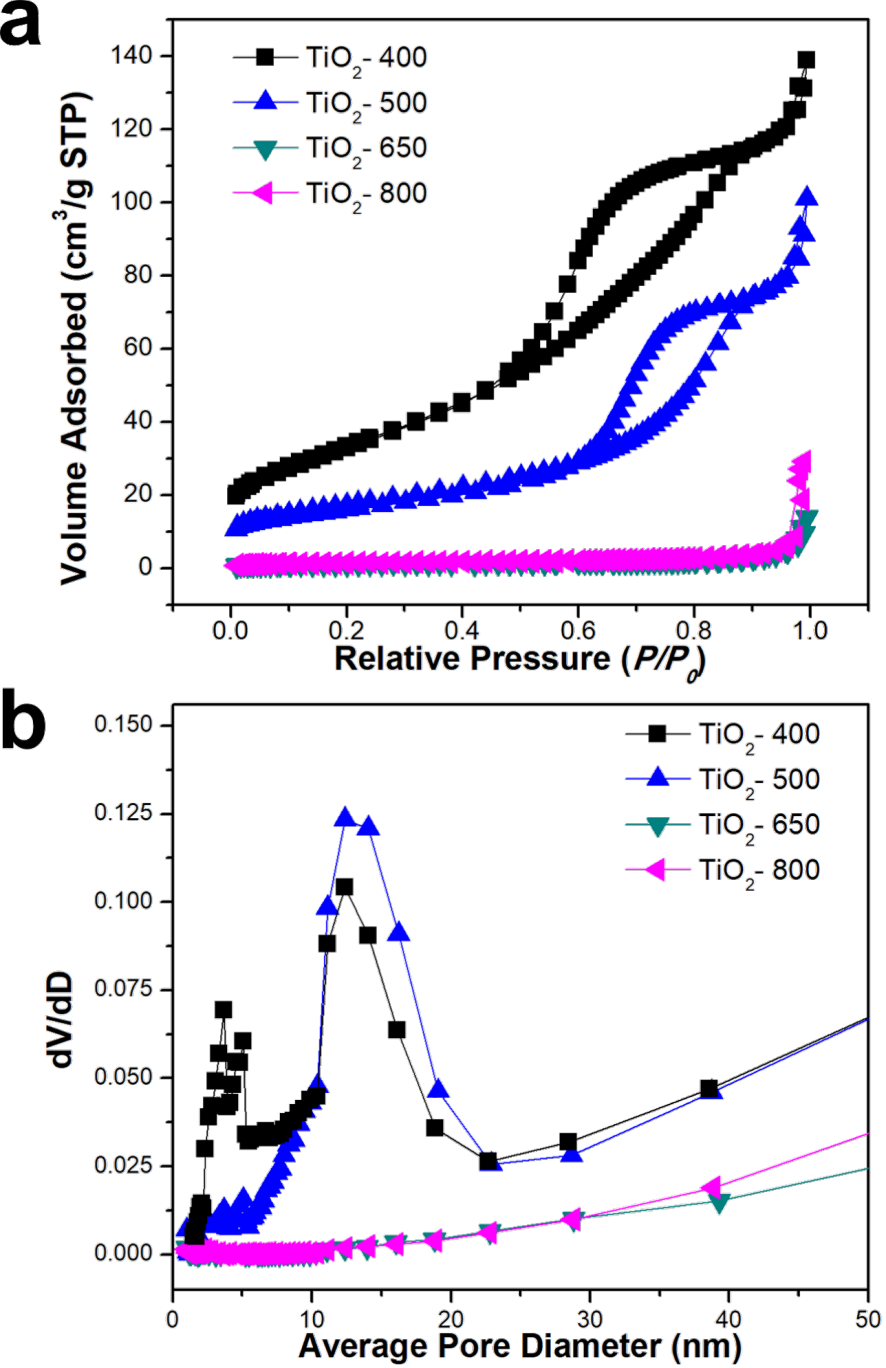
(a) N_2_ adsorption/desorption isotherm and (b) pore size distribution of TiO_2_ particles calcined at different temperatures (≈400–800 °C).

We also investigated the pore characteristics of the TiO_2_ samples calcined at different temperatures. Figure 5 provides the nitrogen adsorption/desorption isotherm and corresponding Barrett–Joyner–Halenda (BJH) pore size distributions of the TiO_2_ samples. The TiO_2_ sample calcined at 400 °C (TiO_2_-400) displayed a typical type IV isotherm with a well-developed hysteresis loop that indicated mesoscale porosity (Figure 5a). The TiO_2_ sample (TiO_2_-400) calcined at a relatively low temperature (400 °C) had a large adsorption capacity in the range of monolayer adsorption (0.1 < *P*/*P*_0_ < 0.25), which indicated highly porous structures with a large BET surface area. However, the amount of monolayer adsorption of the TiO_2_ samples continuously decreased, which indicated decreasing porosity and BET surface area, as the calcination temperature increased. When the TiO_2_ samples were calcined at temperatures greater than 650 °C, they showed negligible N_2_ adsorption volumes over all of the *P*/*P*_0_ range, indicating a small BET surface area (Figure 5a). The measured surface area for the TiO_2_ samples calcined at 400, 500, 650, 800 °C were 123, 62, 5 and 5 m^2^/g respectively. Figure 5b provides the pore size distribution of the TiO_2_ sample calcined at different temperatures as calculated by the BJH method using the adsorption branch of the N_2_ adsorption isotherm. The TiO_2_ particles calcined at 400 °C displayed an obvious distribution in the range of 0–5 nm with a peak in the range of 10–25 nm, which indicated the presence of a mesopore. When the TiO_2_ particle was calcined at 500 °C, it exhibited an obvious peak in the range of 10–25 nm, although there were no sharp peaks in the BJH pore size distribution curves. However, once the TiO_2_ sample was calcined at high temperatures, e.g. more than 650 °C, they did not show any obvious peaks in the range related to mesopores, which indicated an almost non-porous characteristic.

It is well-known that the amorphous TiO_2_ layer has a non-porous or microporous characteristic [14,26]. As the calcination temperature was increased, it should be noted that the amorphous TiO_2_ layer was locally aggregated, fused and crystallized into small grains which resulted in the appearance of mesoporosity. When the TiO_2_ samples were subjected to calcination at high temperatures, small TiO_2_ crystallites were grown, severely fused and finely shirked, which resulted in decreased mesoporosity and a small BET surface area but increased crystallinity.

To estimate the carbon content and thermal behavior of the TiO_2_ samples, TGA analysis was conducted. Since only a surfactant (HPC), titanium precursor (TBOT) and solvent were used during the synthesis, the final TiO_2_ sample should mainly consist of elements such as Ti, O, H and C, respectively. Therefore, weight loss during a thermogravimetric analysis (TGA) experiment originates from desorption of water, dihydroxylation and the combustion reaction of carbon contents. The TGA analysis was conducted using the surfactant HPC as a reference run to monitor the decomposition temperature (Supporting Information File 1, Figure S3a). It should be noted that HPC is dramatically decomposed in the range 300–500 °C. Amorphous TiO_2_ and the TiO_2_-350 sample showed steep weight loss in the range of ambient temperature to ≈150 °C, which is attributed to desorption of water, and continuous loss in the range of 300 to 500 °C, indicating the thermal oxidation and decomposition of HPC and carbon remained in the particle. However, TiO_2_-500 and TiO_2_-800 showed a negligible weight loss, indicating no obvious carbon species in the particle (Supporting Information File 1, Figure S3b). In addition, we measured the zeta potential of the calcined TiO_2_ samples for estimating the surface charge. Although zeta potential values of TiO_2_ samples do not exactly represent quantitative analysis of surface functional groups, it is a useful method to estimate the surface charge which is highly related with the surface OH group. The results show that the zeta potential values are estimated to be approximately −40, −23.8 and −24.9 mV nm for TiO_2_-350, TiO_2_-500, and TiO_2_-800, respectively. It means that the TiO_2_ sample calcined at low temperature (350 °C) should have a relatively large amount of surface OH groups. Once the calcination temperature is higher than a certain point (e.g., 500 °C), the zeta potential values of the TiO_2_ samples (TiO_2_-500 and TiO_2_-800) are almost similar, indicating the presence of a similar number of surface OH groups.

**Figure 6:**
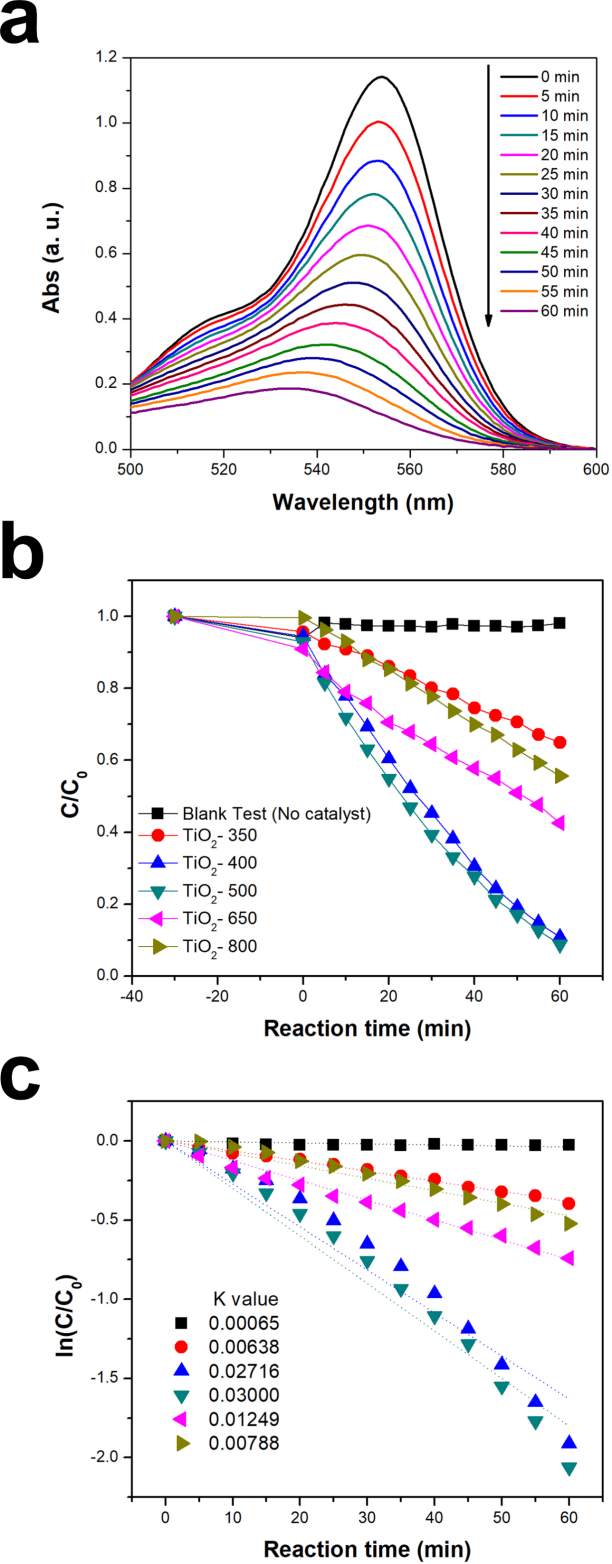
(a) UV–vis absorption spectra indicating degradation of rhodamine B (RhB) using the TiO_2_-500 sample, (b) photocatalytic degradation of RhB under UV–vis light irradiation and (c) semilogarithmic plot versus time for the different TiO_2_ samples: Blank Test (no catalyst) (■), TiO_2_-350 (●), TiO_2_-400 (▲), TiO_2_-500(▼), TiO_2_-650 (◄) and TiO_2_-800 (►).

The photocatalytic activity of the prepared TiO_2_ samples was evaluated by monitoring the degradation of rhodamine B (RhB) under UV–vis light irradiation. Figure 6a shows the typical absorption spectra change for an aqueous RhB solution after UV–vis light irradiation for constant time intervals using TiO_2_ particles (TiO_2_-500) calcined at 500 °C. The strong absorption peak at 553 nm continuously decreased and finally disappeared after 60 minutes. This indicated that our TiO_2_ particles photo-chemically degraded the RhB molecule completely under UV–vis light irradiation conditions. The photocatalytic performance of the TiO_2_ sample towards RhB degradation, determined by monitoring the peak intensity change at 553 nm vs time, was summarized in Figure 6b. Before UV-light irradiation, the reaction mixture containing the catalyst and RhB was stirred for 30 min to ensure saturation of the RhB adsorption on the surface of the TiO_2_ particles. All of the TiO_2_ catalysts showed a similar adsorption capacity in the range of ≈9% of *C*/*C*_0_. In the blank experiment (i.e., no catalyst), the *C*/*C*_0_ of RhB was decreased by only ≈2% after UV–vis irradiation for 60 min, while the degradation of RhB significantly improved when the TiO_2_ catalysts were used. The TiO_2_ sample calcined at 350 °C (TiO_2_-350) exhibited the lowest catalytic activity among all of the catalysts employed in this study. When the TiO_2_ sample was calcined at 400 °C (TiO_2_-400), it showed a dramatically enhanced catalytic activity. In particular, the TiO_2_ sample calcined at 500 °C (TiO_2_-500) showed the best performance among the TiO_2_ samples tested in this study. The relative photocatalytic activity of the TiO_2_ samples for RhB degradation were as follows: TiO_2_-500 ≥ TiO_2_-400 > TiO_2_-650 > TiO_2_-800 ≥ TiO_2_-350.

In order to estimate the first-order reaction rate constant and compare reaction kinetics, Figure 6c displays the data in semi-logarithmic form. The blank test (i.e., no catalyst) result showed a negligible rate constant (0.00065 min^−1^). The TiO_2_-350 sample showed the lowest *k* value among all of the TiO_2_ samples. As the calcination temperature was increased up to 500 °C, the *k* value continuously increased. The TiO_2_-500 sample had the highest *k* value (0.03 min^−1^), which indicated the highest photocatalytic activity. As the calcination temperature was further increased, the *k* values were reduced, which indicated a decrease of photocatalytic efficiency.

In order to further ensure the relationship between photocatalysis performance and calcination temperature, we carried out calcination at different temperatures using other TiO_2_ particles which were prepared in pure ethanol conditions as presented in Figure 1a. Similar to the results presented in Figure 4, when a sample was calcined at different temperatures, the resulting TiO_2_ particles showed different crystalline phases and degrees of crystallinity. Especially, when the TiO_2_ sample prepared in pure ethanol solvent was calcined at 500 °C, it showed sharp anatase peaks with a weak (110) diffraction peak of rutile phase (Supporting Information File 1, Figure S4), indicating that the sample has a mixed phase of well-crystallized anatase with minor rutile. In addition, we also tested the photocatalytic activity of the TiO_2_ particles which were prepared in pure ethanol solvent conditions followed by calcination at different temperatures. As shown in Supporting Information File 1, Figure S5, The TiO_2_ sample calcined at 500 °C showed the highest photocatalytic activity compared to other TiO_2_ samples calcined at either 350 or 800 °C.

We also investigated the effect of other synthesis parameters, such as change in the HPC concentration, on the photocatalytic performances of TiO_2_ samples. We calcined the TiO_2_ samples prepared using different HPC concentrations with a fixed calcination temperature (500 °C). When the as-synthesized TiO_2_ samples prepared using different HPC conditions were calcined at 500 °C, all samples showed the mixed crystalline phase of well-developed anatase with minor rutile (Supporting Information File 1, Figure S6a). Thus, it should be noted that the crystalline properties of all TiO_2_ samples calcined at same temperature (500 °C) are similar even though different amounts of surfactant are used during the synthesis steps. However, the use of different HPC concentrations can eventually affect the pore-forming properties (such as pore size and surface area) of the final TiO_2_ particles. When a relatively large amount of HPC was used during the synthesis, the final TiO_2_ sample showed a large surface area. The samples TiO_2_(HPC-1666.67)-500 and TiO_2_(HPC-416.67)-500 showed the most well-developed mesoporous structure with a high surface area compared to the other TiO_2_ samples including TiO_2_(HPC-166.67)-500, TiO_2_(HPC-41.67)-500 and TiO_2_(HPC-0)-500 (Supporting Information File 1, Figure S6b). The calculated BET surface area of TiO_2_ samples prepared using 1666.67, 416.67, 166.67, 41.67 and 0 mg/L of HPC were ≈43, 62, 12, 20 and 0.7 m^2^/g, respectively.

Supporting Information File 1, Figure S7 compares the photocatalytic performance of TiO_2_ samples prepared using different concentrations of HPC followed by calcination at a fixed temperature of 500 °C. The samples TiO_2_(HPC-1666.67)-500 and TiO_2_(HPC-416.67)-500 showed relatively fast RhB degradation kinetics compared to the other samples. Considering that all of the TiO_2_ samples have similar crystalline properties (i.e., all samples have a well-developed anatase crystalline structure with minor rutile phase), the performance difference likely originates from differences in the surface area. It is well known that the photocatalytic activity is highly influenced by several factors, such as crystalline properties, surface area, dispersity, etc. Since we calcined all TiO_2_ samples at the same temperature, it can be regarded that the crystalline properties and surface properties are similar. However, different concentrations of surfactant used during the synthesis affect the surface area of the final TiO_2_ samples, resulting in different photocatalytic activity. Based on our observations, we can conclude that our TiO_2_ particles, having high anatase crystallinity with mixed phase as well as a large surface area, should have beneficial effects including light absorption, charge separation and facile surface reaction, all resulting in the enhanced photocatalytic efficiency towards RhB degradation.

As pointed out in the Introduction section and in our previous study, many factors play an important role in determining the photocatalytic activity, such as high crystallinity with the right crystalline phase and large surface area [13,15]. In this study, as the calcination temperature was increased up to 400 °C, amorphous TiO_2_ was converted to a metastable anatase phase. When the calcination temperature was increased even higher (e.g., 500 °C), the anatase phase grew and transformed into crystalline rutile. As the calcination temperature was increased up to 800 °C, the anatase phase continuously transformed to rutile and only the rutile phase could be observed. Although the high calcination temperature leads to an increase of crystallinity with the controllable crystalline phase, it resulted in a significantly small surface area. In this study, when the TiO_2_ sample was calcined at 500 °C (TiO_2_-500), it eventually showed high anatase crystallinity with the mixed crystalline phase of anatase and rutile and it had a significantly higher surface area of ≈61 m^2^/g. As previously mentioned, the outstanding catalytic performance of commercial P25 TiO_2_ is mainly attributed to the mixed phase composition of anatase and rutile, which might have a beneficial effect on light absorption and charge separation [20]. It should be noted that the increased activity of P25 TiO_2_ is due to the electron sink of rutile preventing electron–hole recombination in the anatase phase. It allows an anatase-originating hole to move to the surface, resulting in a high surface reaction under UV light [34]. Another hypothesis regarding the exceptional activity of anatase and rutile P25 is that the presence of rutile crystallites generates a favorable structure in which rapid electron transfer from rutile to lower energy lattice trapping centers of anatase phase occurs under visible-light irradiation. This leads to a more stable charge separation and allows holes to reach the surface for redox reactions. In addition, the small band gap of the rutile phase extends the useful range of light energy into the visible light spectrum [20]. Although our TiO_2_-500 catalyst does not have the same dimensions and particle shape, it has a mixed phase of well-crystallized anatase with rutile, which is like highly active P25 TiO_2_. Even though the exact functions of rutile phase in mixed phase materials is still controversial in photocatalysis, our TiO_2_-500 catalyst should have similar beneficial effects on light absorption and charge separation by high anatase crystallinity with mixed phase composition as well as an additional large surface area, which would result in the enhanced photocatalytic efficiency towards RhB degradation.

## Conclusion

We demonstrated the general synthesis of uniform TiO_2_ colloidal particles with controllable properties and enhanced photocatalytic activity towards RhB degradation. The particle synthesis was conducted in mixed solvent conditions by sol–gel chemistry through a one-pot process followed by calcination. This study allowed for the systematic investigation of the influences of synthetic parameters such as a solvent composition, surfactant concentration, and concentration of the titanium butoxide. Under the optimum ratio of mixed solvent conditions (ethanol/acetonitrile 3:1), uniform and monodisperse colloidal TiO_2_ particles were obtained. As the concentration of surfactant was decreased and the concentration of the TiO_2_ precursor was increased, the size of the TiO_2_ particles increased. Tuning the calcination temperature can control the crystallinity and crystalline phase of the colloidal TiO_2_ particles. As the calcination temperature was increased and the anatase crystalline phase transformed to its rutile counterpart, the surface area correspondingly decreased. This study determined the optimum crystallinity and surface area that yields the highest photocatalytic performance towards RhB degradation under UV–vis irradiation. Our systematic investigation may provide a good example for elucidating a rational design for more efficient colloidal TiO_2_-based photocatalysts.

## Experimental

**Materials and Chemicals**: Ethyl alcohol (C_2_H_5_OH, 99.9%, anhydrous), acetonitrile (ACN, CH_3_CN 99.9%, special guaranteed grade) and ammonium hydroxide (NH_4_OH, 28%) were obtained from Daejung Chemical Company. Hydroxypropylcellulose (HPC, MW ≈80,000) and titanium(IV) butoxide (TBOT, 97%, reagent grade) were obtained from Aldrich. Rhodamine B (RhB, 95%, HPLC grade) was purchased from Sigma-Aldrich. All chemicals were used as received without further treatment.

**Synthesis**: Uniform spherical TiO_2_ particles were prepared via a sol–gel reaction of titanium alkoxide in a mixed solvent solution in the presence of a surfactant and an aqueous ammonia solution. The desired amount of hydroxypropylcellulose (HPC) was dissolved in a solution of ethanol and acetonitrile with different volume ratios under vigorous stirring. After completely dissolving the HPC, aqueous ammonia (0.8 mL) was added to the solution. After stirring for 10 min, a solution of tetrabutyl titanate (TBOT) with the desired amount in a mixture of ethanol and acetonitrile was quickly injected into the above solution. The total volume of the reaction mixture was ≈120 mL. The mixture was stirred for 2 h under ambient conditions and the white precipitate was isolated by centrifugation. The white precipitate was washed with ethanol and with DI water a couple of times and dried under vacuum to obtain amorphous spherical TiO_2_ particles. The dried TiO_2_ particles were charged into an alumina boat in a tubular furnace and calcined at the desired temperature for 3 h under air conditions. After calcination under air, the amorphous TiO_2_ particles were crystallized to either the anatase or rutile phase. Before the photocatalytic reaction experiment, the calcined sample (30 mg) was treated with an aqueous NaOH solution (10 mL, 2 M) to improve dispersity in aqueous solution.

**Characterization**: The particle morphology and dispersity were observed using transmission electron microscopy (TEM, JEM-2100, JEOL). The crystalline phases of the samples were determined by X-ray diffraction (XRD) analysis using a Rigaku D/mas-2200 diffractometer with Cu Kα radiation (λ = 1.5406 Å). The pore property was characterized using the nitrogen adsorption/desorption technique at 77 K using a nitrogen sorption instrument (ASAP 2000, Micromertics).

**Catalytic activity test:** The photocatalytic activity of the prepared samples was evaluated by following the photocatalytic degradation of rhodamine B. Before the photocatalysis test was initiated, the well-dispersed catalyst in the solution was first irradiated under UV light for 30 min to remove any residual organic contaminates on the surface of the catalyst. The catalyst (30 mg of TiO_2_) was dispersed in an aqueous RhB solution (50 mL, 10^−5^ M) in a 100 mL reactor cell and the solution was stirred in dark conditions for 30 min to ensure the adsorption of RhB onto the surface of the catalyst. A 70 W commercial LED lamp was used as the light source during the photocatalysis experiments. The concentration of RhB was monitored by UV–vis spectrophotometry (Thermo Fisher Scientific, Genesys 10S) during the reaction.

## Supporting Information

File 1 Additional experimental results.
