# Supplementary material for: Controllable one-pot synthesis of uniform colloidal TiO2 particles in a mixed solvent solution for photocatalysis
Source: Beilstein J Nanotechnol. 2018 Jun 8;9:1715–27. doi: 10.3762/bjnano.9.163 (PMC6009296; doi:10.3762/bjnano.9.163)
Supplement: File 1 — Additional experimental results. [file Beilstein_J_Nanotechnol-09-1715-s001.pdf]

Supporting Information

for

Controllable one-pot synthesis of uniform colloidal TiO_2 particles in a mixed solvent solution for photocatalysis

Jong Tae Moon[1], Seung Ki Lee[2] and Ji Bong Joo[1*]

Address: [1]Department of Chemical Engineering, Konkuk University, 120 Neungdong-ro, Gwangjin-gu, Seoul 05029, Republic of Korea and [2]Department of Life Science, University of Seoul, 163 Seoul Siripdae-ro, Dondaemun-gu, Seoul 02504, Republic of Korea

Email: Ji Bong Joo - jbjoo@konkuk.ac.kr

* Corresponding author

Additional experimental results

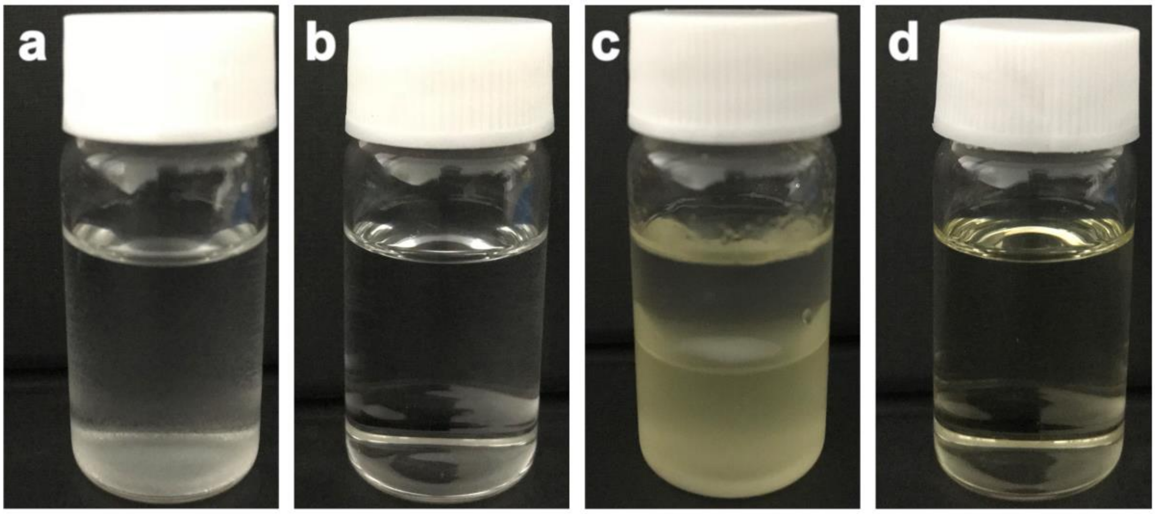

Figure S1. Digital photo images indicating solubility of HPC and TBOT in different solvent. (a) 10 mg of HPC in 10 mL of ACN, (b) 10 mg of HPC in 10 mL of ethanol (c) 5 mL of TBOT in 5 mL of ACN, and (d) 5 mL TBOT in 5 mL of ethanol.

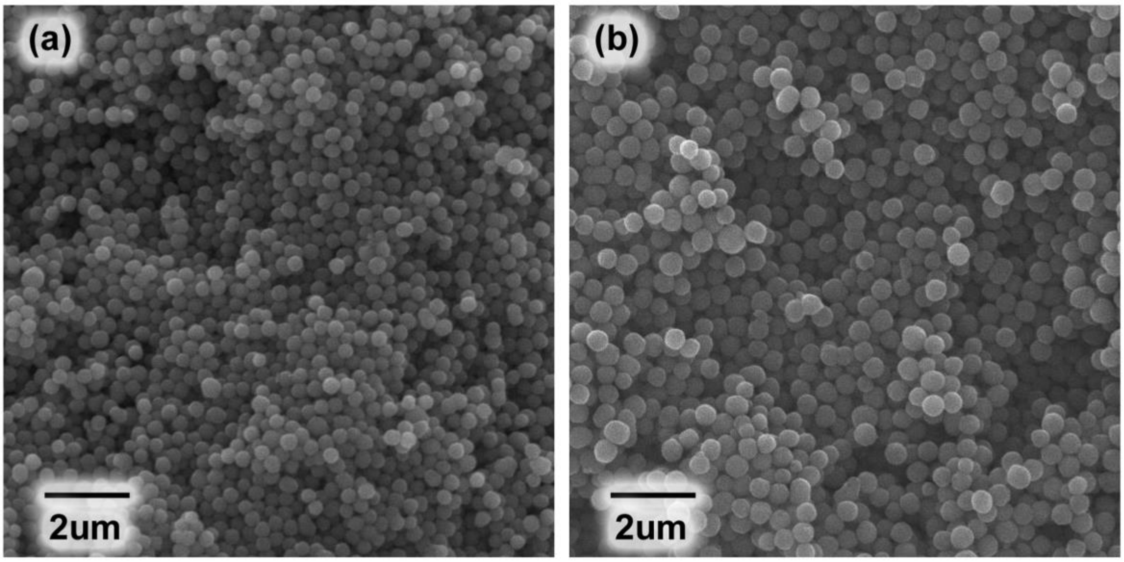

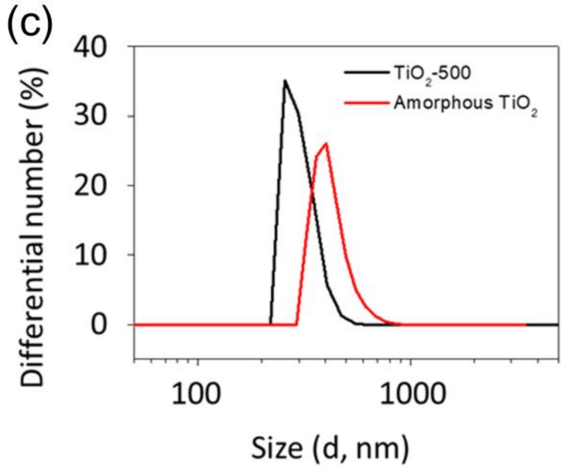

Sample	Size
**Amorphous TiO_2**	347±75 nm
**TiO_2-500**	262±7 nm

Figure S2. (a-b) SEM images, (c) DLS results and Table indicating the average particle diameter of as-synthesized amorphous TiO_2 particles prepared by using when volume ratio of ethanol to ACN as 3:1 (b), and as-calcined one after calcination at 500 ^{o}C (a).

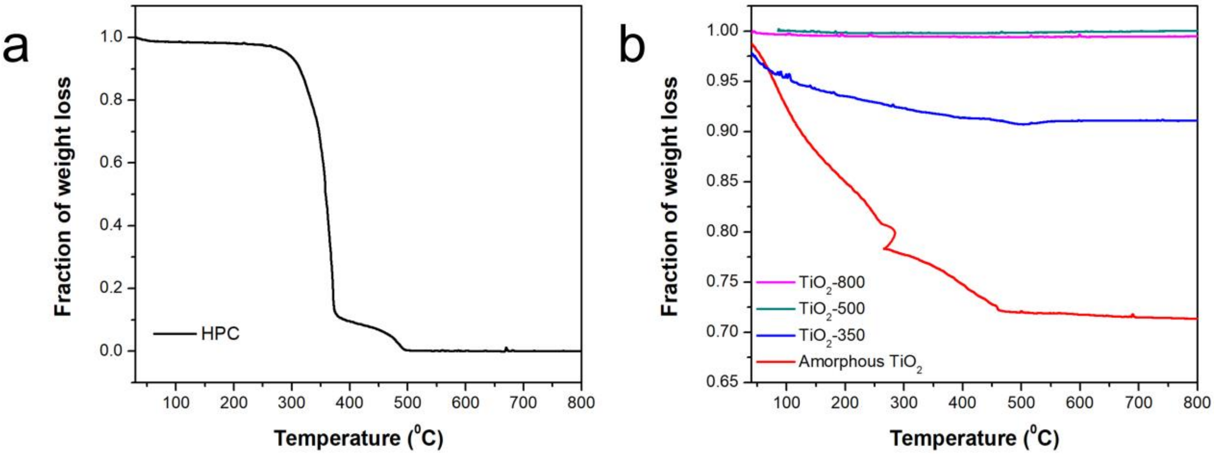

Figure S3. TGA results of (a) HPC decomposition as a reference and (b) TiO_2 samples prepared in this work under air conditions.

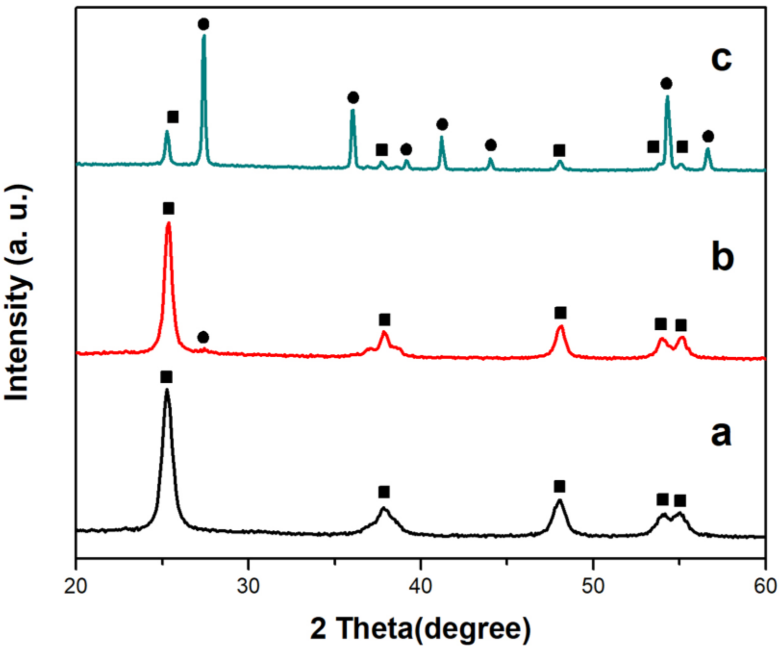

Figure S4. XRD patterns of TiO_2 samples prepared in pure ethanol conditions followed by calcination at different temperatures :(a) 350 (b) 500 and (c) 800 ^{o}C (■ and ● denote anatase and rutile phases, respectively)

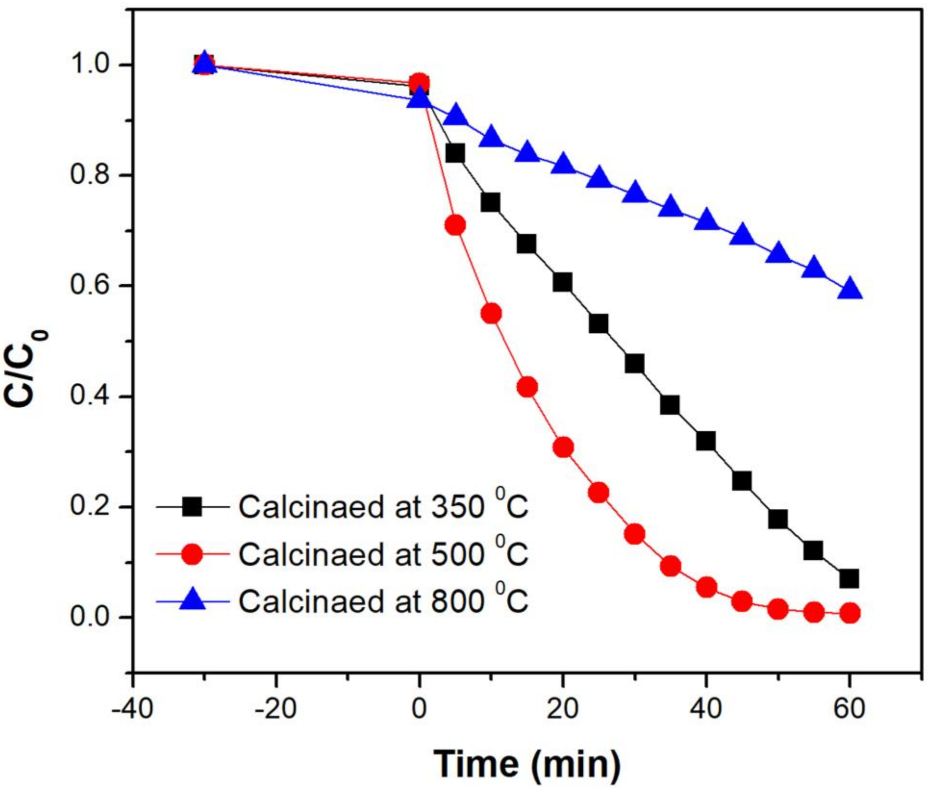

Figure S5. Photocatalytic degradation of RhB under UV-vis light conditions in the presence of TiO_2 samples prepared in pure ethanol conditions followed by calcination at different temperatures.

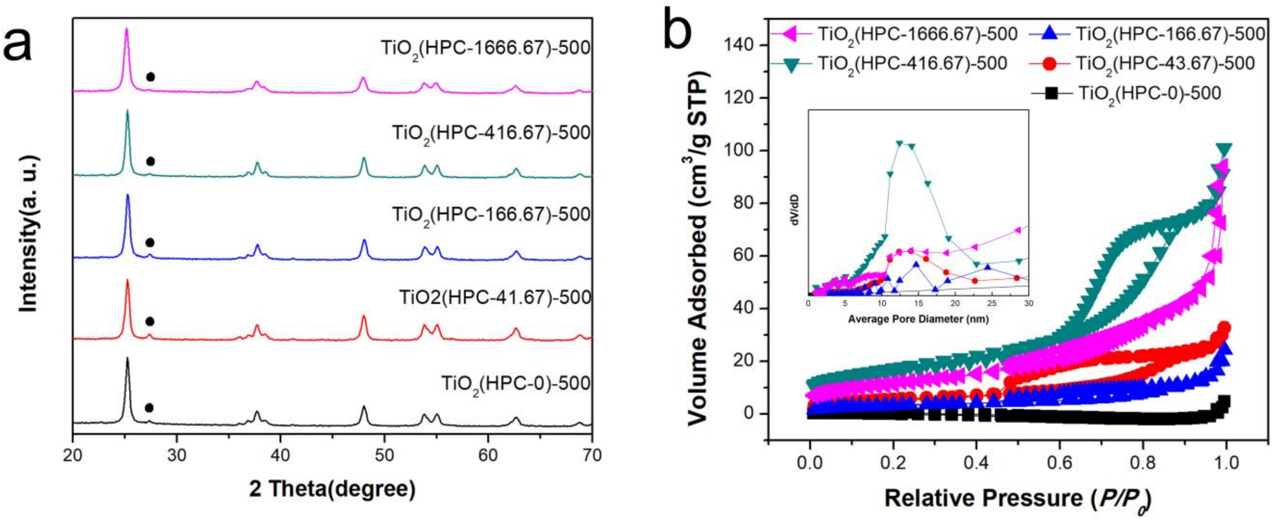

Figure S6. (a) XRD patterns and (b) N_2 isotherms with corresponding pore size distributions of TiO_2 samples prepared by using different concentrations of HPC in the mixed solvent conditions followed by calcination at 500 ^{o}C (● denotes rutile phases)

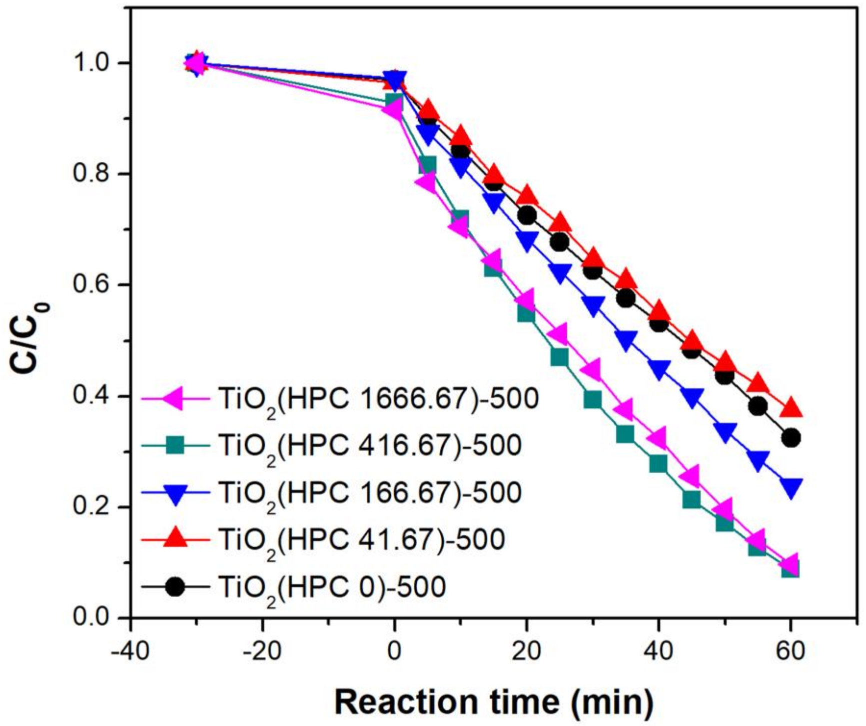

Figure S7. Photocatalytic degradation of RhB under UV-vis light conditions in the presence of TiO_2 samples prepared by using different concentrations of HPC in the mixed solvent conditions followed by calcination at 500 ^{o}C
